# Acute diffuse edematous-hemorrhagic Epstein–Barr virus meningoencephalitis: A case report

**DOI:** 10.1097/MD.0000000000018070

**Published:** 2019-12-20

**Authors:** Jingzhe Han, Zhilei Kang, Yanan Xie, Hui Li, Haiyan Yan, Xueqin Song

**Affiliations:** a Department of Neurology; b Department of MRI, Harrison International Peace Hospital, Hengshui; c Department of Angiocardiopathy; d Department of Neurology, The Second Hospital of Hebei Medical University; e Institute of Cardiocerebrovascular Disease; f Neurological Laboratory of Hebei Province, Shijiazhuang, Hebei, China.

**Keywords:** EBV, meningoencephalitis, next-generation sequencing

## Abstract

**Introduction:**

In this study, we presented a rare case of Epstein–Barr virus (EBV) meningoencephalitis presented with meningoencephalitis-like symptoms and diffuse edematous hemorrhage.

**Patient concerns:**

A 77-year-old male patient was admitted to our hospital with fever, headache, confusion, and unconsciousness for 7 days. Physical examination revealed unconsciousness and stiffness of the neck.

**Diagnosis:**

The final diagnosis was EBV meningoencephalitis.

**Interventions:**

Ganciclovir (two times 350 mg/day, 21 days), methylprednisolone sodium succinate (120 mg, 5 days), and IV immunoglobulins (IV Ig) (0.4 g/kg, 5 days) were given to this patient.

**Outcomes:**

But the patient's clinical symptoms did not improve, and he was still in a coma. His family refused to be further diagnosed and discharged. After discharge for 2 months, the patient was in a coma. Four months later, the patient died of complications of pulmonary infection.

**Conclusion:**

The patient is an adult, and imaging was dominated by intracranial diffuse microhemorrhage and edema, which was different from the typical imaging characteristics of EBV encephalitis as previously reported. This specific imaging change may provide new clinical value for the diagnosis of EBV encephalitis.

## 1 Introduction

Epstein–Barr virus (EBV) is associated with some complications of the central nervous system, such as meningitis, transverse myelitis, cerebellitis, and encephalitis.^[1]^ In patients with normal immune function, EBV-induced meningoencephalitis is a mild self-restricted disease that usually recovers completely.^[2]^ As far as we know, there are few cases of EBV hemorrhagic encephalitis reported in the literature, and the hemorrhage areas are mostly limited to the frontal lobe, parietal lobe, and cerebellum.^[3]^

Magnetic resonance imaging (MRI) can display small or multiple central nervous system injuries more clearly, help doctors diagnose quickly and develop more effective treatment strategies.^[4,5]^ Diffusion weighted imaging (DWI) sequence recognizes lesions of the central nervous system earlier than T2W or FLAIR imaging.^[5–9]^ Next-generation sequencing (NGS) is a potentially revolutionary pathogen identification method, including rare and newly identified viruses,^[10]^ and NGS technology can conduct comprehensive detection of pathogens in CSF samples.^[11]^

In this study, we presented a rare case of EBV meningoencephalitis in an old male patient presented with meningoencephalitis-like symptoms and diffuse edematous hemorrhage in cerebral and cerebellar cortex on MRI, which is different from the typical imaging features of EBV encephalitis in the past, and his disease was also confirmed by NGS.

## 2 Case presentation

**Figure 1:**
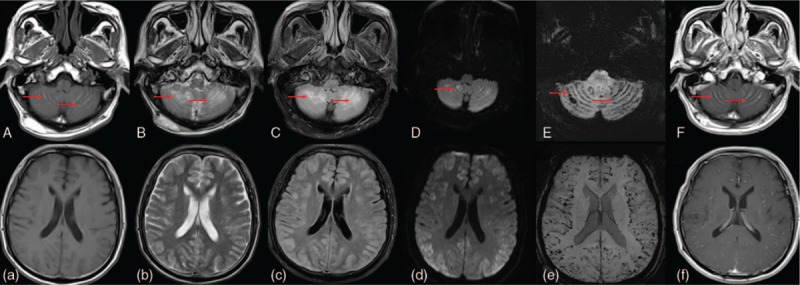
The MRI findings of the patient: **(**A, a) T1 sequence showed cerebellar sulcus short T1 signal, indicated bleeding (arrowhead). **(**B, b**)** T2 showed the long T2 signal of the cerebellum in the focus (arrowhead) and diffuse cerebral cortex swelling; **(**C, c**)** flair showed the abnormal signal of the cerebellar cortex (arrowhead) and diffuse cerebral cortex swelling. **(**D, d**)** DWI in the cerebellar cortex point flake diffusion limited (arrowhead) and diffuse cerebral cortex swelling, cytotoxic edema was suggested. **(**E, e**)** SWI showed diffuse point line like low signal (arrowhead) and diffuse punctate low signal in the cerebral cortex, and extensive haemorrhage was suggested. **(**F, f**)** Enhanced MRI showed cerebellar hemispherical linear enhancement (arrowhead) and diffuse leptomeningeal enhancement, and meningeal involvement was indicated.

A 77-year-old male patient was admitted to our hospital with a 7-day history of fever, headache, mental disorder, and unconsciousness. Physical examination revealed unconsciousness and neck stiffness. No special personal history or family history. His vital signs were: body temperature 37.8°C; heart rate 96 beats/min; respiratory rate 20 breaths/min; BP 138/72 mm Hg. The GCS score was 6 points. Coagulation routine, liver and kidney function, electrolyte, blood glucose hematomy were not abnormal, HIV antibody negative. No abnormalities in immune and tumor markers. Blood routines showed a lymphocyte ratio of 14.8%. Lumbar puncture showed that pressure was greater than 350 mm H_2_O. CSF protein was 4098 mg/L associated with pleocytosis (38 cells/mL), but the glucose and chlorides tests were normal. Cytology examination of cerebrospinal fluid (CSF) showed that lymphocytes were dominated and the number of activated monocytes increased, and several erythrocytes could be seen, without the appearance of atypical cells and cryptococcus neoformans. CSF culture was negative for both bacteria and fungi. Antibodies of autoimmune encephalitis in blood and CSF were negative. Magnetic resonance imaging was performed on the third day of admission. MR parameters: diffusion sensitivity factor B was 0 and 1000 s/mm^2^, layer thickness 6 mm, spacing 1.2 mm, and matrix 256 ∗ 256. The scanning parameters were as follows: sagittal T1WI (TR2060 ms/TE11 ms); axial T2WI (TR4000 ms/TE101 ms); T1WI (TR2340 ms/TE980 ms); Flair (TR8000 ms/TE94 ms); and DWI (TR3000 ms/TE68 ms). SWI (TR27 ms/TE20 ms) was 1.2 mm thick and scanned in 3D. The T1 sequence showed a short T1 signal in the cerebellum groin, indicating bleeding. The T2 sequence showed the cerebellum long T2 signal lesions and diffuses cerebral cortex swelling. Flair showed high signals in the cerebellum cortex and diffuse swelling of the cerebral cortex. DWI showed limited microcephaly and diffuse cerebral cortex swelling, suggesting cytotoxic edema. SWI showed diffuse dot-line-like low signals in the cortex of the cerebellum and the cerebral cortex, suggesting extensive micro-bleeding. Enhanced MRI showed cerebellum line-like reinforcement and diffuse flexor meninges reinforcement, suggesting that the meninges are affected. (Fig. 1) Head MRA revealed mild arteriosclerosis. 24-hour ambulatory EEG showed diffuse 2 to 3 Hz waves, with a 20 to 40 V amplitude. EBV–DNA was detected by NGS detection of CSF, then EBV meningoencephalitis was highly suggested. The EBV–polymerase chain reaction (PCR) of CSF showed that the copy number of EBV–DNA was 22,100 copies/mL, the EBV meningoencephalitis was finally diagnosed.

The patient was treated with ganciclovir (two times 350 mg/day, 21 days), methylprednisolone sodium succinate (120 mg, 5 days) and IV immunoglobulins (IV Ig) (0.4 g/kg, 5 days). Twenty days after admission, his lumbar puncture pressure was 200 mm H_2_O, CSF protein was 986 mg/L associated with pleocytosis (18 cells/mL), the glucose and chlorides were normal. Thirty days after admission, his lumbar puncture pressure was 180 mm H_2_O, CSF protein was 442 mg/L associated with pleocytosis (10 cells/mL), but his clinical symptoms did not improve, he was still in a coma. The patient was hospitalized for 32 days. His family refused further medical treatment and discharged from the hospital. Two months after discharge, the patient was still in a coma. Four months later, the patient died of pulmonary infection complications.

## 3 Discussion

The pathogenesis of EBV encephalitis may be due to either direct central nervous system (CNS) EBV invasion or a postinfectious inflammatory response mediated by antineuronal antibodies.^[12,13]^ The diagnosis of EBV encephalitis based on the clinical features and supported with findings on imaging and laboratory testing. The DWI sequence provides image contrast that relies on the movement of water molecules, which is important in cases of increased or limited diffusion, such as ischemic stroke, intracranial infection, or trauma.^[4–7]^ The infection of the nervous system is characterized by changes in the diffusion of water caused by cytotoxic edema, which can be detected by DWI. It has been reported that DWI is usually the first sequence activated by cytotoxic cortical edema in necrotic tissues of EBV encephalitis.^[5–9]^ The main manifestations of the patient were headache, unconsciousness, and fever, and the patient's DWI presented high roaming signals in the epidermal cortex (dispersal restriction), indicating toxic edema caused by viral infections.

The differential diagnosis of EBV mainly includes high hemolytic ammonia, hypoglycemic encephalopathy, epilepsy persistence, hypoxia encephalopathy,^[1,14,15]^ etc, these symptoms can be excluded from this patient. Susceptibility Weighted Imaging (SWI) in this patient indicated diffuse and dot like low signal in cerebellum and cerebral cortex, where diffuse intracranial microhemorrhage and edema were the main imaging features, which is associated with the major histopathological patterns of EBV edematous-hemorrhagic encephalitis.^[1]^ Several red blood cells can be seen after extracytological CSF puncture injury, which also supports this judgment. It has been reported that because of the special affinity of EB virus to basal ganglia, high signal changes in bilateral striatum, thalamus and cortex can be seen on weighted MRI-T2 images.^[16]^ The involvement of white matter and brainstem corpus callosum pressure has also been reported,^[15,1]^ but it is very rare. In this case, basal ganglia were not involved, but corticocerebellar hemisphere was mainly involved with diffuse hemorrhage, which was inconsistent with the previously reported imaging features.^[3]^

At present, some scholars use GCS score to evaluate the severity of inflammatory injury.^[17,18]^ Clinicians rely more on the results of lumbar puncture, imaging changes and electrophysiological changes to reflect the severity of the disease. The patient's GCS score was 6 points on admission and 7 points on discharge. The improvement was not obvious. The prognosis of EBV encephalitis is different in different patients, from complete recovery to death. Most patients with EBV encephalitis with normal immune function^[3]^ have good prognosis. No evidence of abnormal immune function was found in this patient. Combined with CSF changes, diffuse imaging changes and EEG results, it was suggested that brain tissue injury was serious, considering the poor prognosis of this patient may be related to the extent of damage to brain tissue by EBV.

Epstein–Barr virus infection of the nervous system may be due to the direct invasion of the nervous system by EB virus, or the immune response after antibody-mediated infection.^[14,12]^ Three histopathological mechanisms of EB virus infection have been reported: edema–hemorrhage; perivascular mononuclear cell infiltration and virus encapsulation in cerebral cortex cells; inflammatory response of perivascular lymphocyte and demyelinating lesion of white matter (acute infection is associated with autoimmune response).^[19]^ Hemorrhagic encephalitis caused by EB virus infection is rare and lethal. Hemorrhagic edema caused by EB virus encephalitis is mainly seen in primary infection or in children.^[20]^

The main highlight of this case is its typical imaging manifestations, the patient is an adult, and imaging was dominated by meningoencephalitis-like symptoms and diffuse intracranial microhemorrhage and edema, which was different from the typical imaging characteristics of EBV encephalitis as previously reported.^[15,21,22]^ This specific imaging change may provide new clinical value for the diagnosis of EBV encephalitis.

## Author contributions

**Conceptualization:** Jingzhe Han, Zhilei Kang, Xueqin Song.

**Data curation:** Jingzhe Han, Zhilei Kang, Yanan Xie.

**Formal analysis:** Zhilei Kang, Yanan Xie.

**Funding acquisition:** Yanan Xie.

**Investigation:** Jingzhe Han.

**Methodology:** Zhilei Kang, Yanan Xie, Hui Li, Haiyan Yan.

**Project administration:** Jingzhe Han, Hui Li.

**Resources:** Jingzhe Han, Zhilei Kang, Hui Li, Haiyan Yan, Xueqin Song.

**Supervision:** Hui Li, Haiyan Yan.

**Visualization:** Jingzhe Han.

**Writing – original draft:** Jingzhe Han, Zhilei Kang.

**Writing – review & editing:** Xueqin Song.
